# Supplementary material for: EnzML: multi-label prediction of enzyme classes using InterPro signatures
Source: BMC Bioinformatics. 2012 Apr 25;13:61. doi: 10.1186/1471-2105-13-61 (PMC3483700; doi:10.1186/1471-2105-13-61)
Supplement: Addtional file 5 — The Java code to format the data files, evaluate and predict. The file enzml_java_code.tar.gz contains the Java code used to format database data to ARFF and XML formats, to execute cross and train-test (jackknife) evaluations and to record evaluation results to database. More information is included in the readme.txt file and the Javadoc files. The code can be used with a MySQL database. To use a different database software, other JDBC drivers might be required. [file 1471-2105-13-61-S5.gz › java_code/enzml2011/doc/uk/ac/ed/inf/csb/taweka2010/FastVectorUtils.html]

FastVectorUtils


---


|  |  |  |
| --- | --- | --- |
| **Package** \| **Class** \| **Use** \| **Tree** \| **Deprecated** \| **Index** \| **Help** | |  |
| **PREV CLASS**   **NEXT CLASS** | **FRAMES**    **NO FRAMES**     **All Classes** |  |
| SUMMARY: NESTED \| FIELD \| CONSTR \| METHOD | DETAIL: FIELD \| CONSTR \| METHOD |  |


---


## uk.ac.ed.inf.csb.taweka2010 Class FastVectorUtils

```
java.lang.Object
  uk.ac.ed.inf.csb.taweka2010.FastVectorUtils
```

---

``` public class FastVectorUtils extends java.lang.Object ```

Utilities for converting between normal vectors/enumerators to Weka FastVector

**Version:**
:   21 Apr 2010

**Author:**
:   Luna De Ferrari luna.deferrari-at-ed.ac.uk

---

**Constructor Summary**

`FastVectorUtils()`


| **Method Summary** | |
| --- | --- |
| `static weka.core.FastVector` | `fromEnumToFastVector(java.util.Enumeration enumeration)` |
| `static weka.core.FastVector` | `fromSetToFastVector(java.util.Set<java.lang.String> set)`             convert from a set to a Weka FastVector |
| `static weka.core.FastVector` | `fromVectorToFastVector(java.util.Vector<java.lang.String> list)`             convert from list with potentially duplicated entries to a set loaded in a weka fastvector |

**Methods inherited from class java.lang.Object**

`equals, getClass, hashCode, notify, notifyAll, toString, wait, wait, wait`

**Constructor Detail**

### FastVectorUtils

```
public FastVectorUtils()
```


**Method Detail**

### fromEnumToFastVector

```
public static weka.core.FastVector fromEnumToFastVector(java.util.Enumeration enumeration)
```

---


### fromVectorToFastVector

```
public static weka.core.FastVector fromVectorToFastVector(java.util.Vector<java.lang.String> list)
```

:   convert from list with potentially duplicated entries to a set loaded in a weka fastvector

    :   **Parameters:**: `list` - a list **Returns:**: a vector containing the set elements

---


### fromSetToFastVector

```
public static weka.core.FastVector fromSetToFastVector(java.util.Set<java.lang.String> set)
```

:   convert from a set to a Weka FastVector

    :   **Parameters:**: `set` - a set **Returns:**: a vector containing the set elements


---


|  |  |  |
| --- | --- | --- |
| **Package** \| **Class** \| **Use** \| **Tree** \| **Deprecated** \| **Index** \| **Help** | |  |
| **PREV CLASS**   **NEXT CLASS** | **FRAMES**    **NO FRAMES**     **All Classes** |  |
| SUMMARY: NESTED \| FIELD \| CONSTR \| METHOD | DETAIL: FIELD \| CONSTR \| METHOD |  |


---
